# Role of Mass Transfer Phenomena in Electrochemical Nitrate Reduction: A Case Study Using Ti and Ag-Modified Ti-Hollow Fiber Electrodes

**DOI:** 10.1021/acsengineeringau.4c00035

**Published:** 2024-12-24

**Authors:** Ainoa Paradelo Rodríguez, Guido Mul, Bastian T. Mei

**Affiliations:** † Photocatalytic Synthesis Group, Faculty of Science and Technology of the University of Twente, PO Box 217, Enschede 7500 AE, Netherlands; ‡ Technische Chemie, Ruhr-Universität Bochum, Universitätsstr. 150, Bochum 44801, Germany

**Keywords:** electrochemical nitrate reduction, porous titanium electrodes, silver particles, interfacial convective mass transport, ammonium production

## Abstract

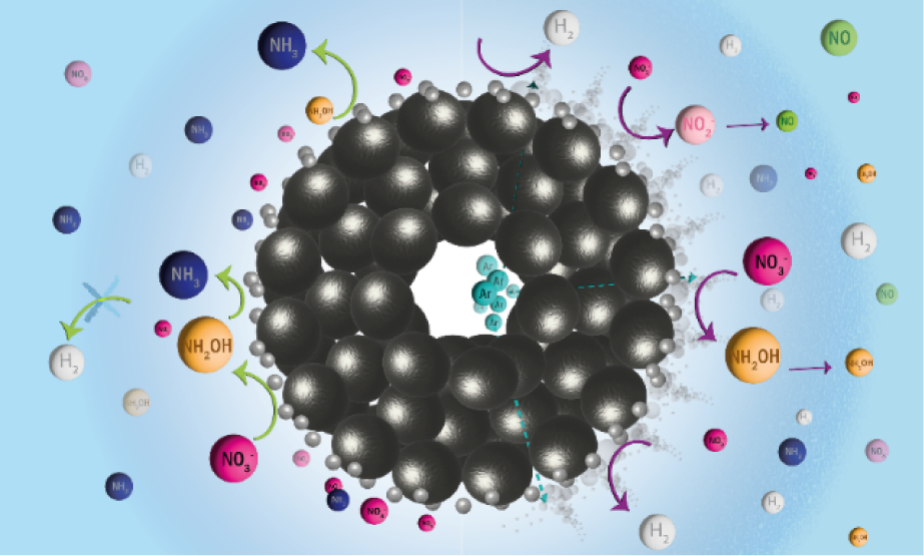

Decentralized electrochemical reduction of nitrate into ammonium is explored as a viable approach to mitigate nitrate accumulation in groundwater. In this study, tubular porous electrodes made of titanium (termed hollow fiber electrodes or HFEs) were successfully modified with silver (Ag) nanoparticles through electrodeposition. Under galvanostatic control and in acidic electrolyte, Ag deposition on Ti HFE resulted in an increase in the Faradaic efficiency for ammonium formation from low concentrations of nitrate (50 mM), but only under reaction conditions of restricted mass transport. For conditions of favorable transport, facilitated by an inert gas flow (Ar) exiting the pores, a higher nitrate conversion but an increase in hydroxylamine selectivity at the expense of the ammonium selectivity are observed for Ti/Ag hollow fiber electrodes. For Ti/Ag electrodes, it is concluded that ammonium formation is prevented by effective removal of surface intermediates. Remarkably, for unmodified Ti hollow fiber electrodes, the Faradaic efficiency to ammonium is significantly improved when operated at high current densities and in conditions of high mass transport. The selectivity to liquid products even surpasses the selectivity of Ti/Ag electrodes. These findings indicate that nitrate reduction to ammonium at Ti and Ti/Ag hollow fiber electrodes can be achieved at comparable rates but under distinctly different process conditions. In fact, for Ti electrodes, operation at a lower applied potential compared to Ti/Ag electrodes is feasible, ultimately resulting in reduced energy consumption. This study thus highlights the importance of controlling the interfacial electrode environment, particularly when comparing and evaluating the effectiveness of electrode materials in electrochemical nitrate reduction. The study also reveals that transport phenomena affect electrode material-dependent activity–selectivity correlations and must be considered in ongoing material development efforts.

## Introduction

Ammonia is an indispensable commodity chemical (about 176 million tons produced per year), and around 70% of its global production is used for fertilizers. Ammonia is currently formed by the Haber Bosch process requiring high temperature and pressure leading to annual CO_2_ emissions of about 500 million tons.^1,2^ Another environmental concern is the increasing concentration of groundwater nitrate, mostly attributed to the use of chemically produced fertilizers.^3,4^ Converting nitrate to ammonia (or ammonium) offers an opportunity to address the above issues.

(Electro)catalytic conversion of nitrate has been frequently studied, mainly aiming at selective formation of nitrogen for water purification purposes.^4−8^ Recently, the selective formation of ammonia and ammonium from nitrate sources has received significant attention.^9−13^ Electrochemical reduction of nitrate to ammonia (high pH) or ammonium (low pH) is a challenging reaction requiring the transfer of eight electrons and nine (ammonia) or ten (ammonium) protons.^5,14,15^ Among all metals and bimetallic materials investigated,^16−22^ a favorable selectivity to ammonia/ammonium over a wide range of process conditions has been observed for titanium electrodes.^23−27^ Moreover, availability, relatively low cost, and good corrosion resistance of Ti are considered beneficial.^23^ In acidic conditions, a Faradaic efficiency (FE) to ammonium of 82% (at −1 V vs RHE) was achieved using a relatively high nitrate concentration (0.4 M).^23^ At significantly lower nitrate concentrations (50 mM) using tubular porous Ti electrodes (Ti hollow fiber electrodes), a FE_NH3_ of 58% was still achievable.^24^ Utilizing these hollow fiber electrodes under conditions of effective local mass transport, facilitated by introducing gas-induced mixing using a flow of inert gas exiting the wall of the hollow fiber electrode (flow-through operation), still resulted in a FE_NH3_ of 45%. It was recently shown by a combination of continuum model simulations and in situ infrared absorption spectroscopy that the product selectivity of electrochemical nitrate reduction using Ti electrodes under potentiostatic control depends on the equivalent diffusion layer thickness.^25^ For process conditions favoring the formation of thin equivalent diffusion layer thicknesses, for example, induced by electrolyte flow, a shift toward nitrite is obtained as a result of an increase in interfacial pH caused by the overall higher reaction rate.^25^ It was concluded that relieving mass-transport limitations is beneficial for the nitrate removal rate but detrimental for the selectivity of Ti electrodes in producing ammonium.^24,25^ However, a detailed understanding of flow versus gas-induced mixing has still to be achieved.

In addition to Ti, Ag electrodes were shown to facilitate nitrate conversion to ammonia with appreciable selectivity or FE.^28,29^ Using oxide-derived Ag electrodes, a FE_NH3_ up to 89% was determined for slightly acidic (pH 4) electrolytes.^28^ NO reduction (an important intermediate in the nitrate reduction reaction) using nanostructured Ag electrodes and a metal complex for NO capture was also shown to proceed with close to unity FEs at moderate current densities (50 mA/cm^2^).^30^ Recently, we have demonstrated by electrochemical mass spectrometry (EC-MS) that synergistic effects in nitrate reduction are obtained using bimetallic Ti/Ag electrodes.^31^ Particularly, when Ti plates were modified by Ag particles, the overpotential for nitrate reduction decreased significantly and generally a higher FE_NH3_ compared to the pure Ti or Ag metal electrodes was observed in stirred batch reactors at modest current densities of up to 15 mA/cm^2^.^31^ Thus, exploring the properties of Ag/Ti electrodes, particularly at relevant current densities, is still required.

In this study, we utilized (Ag-modified) porous tubular Ti hollow fiber electrodes to explore the relation between the electrode material and local mass transport induced by gas-induced mixing. Under galvanostatic control, flow-by (using conventional electrolyte purging with a sparger positioned in close proximity to the electrode) and flow-through conditions (enhanced interfacial mixing by using an inert gas flow exiting the wall of the hollow fiber electrode, HFE) were used to investigate the effects of efficient local mixing on the activity and selectivity of Ti and Ti/Ag electrodes in nitrate reduction. We will demonstrate that the effect of flow configuration (flow-by vs flow through operation) on conversion, FE, and selectivity, strongly depends on the composition of the HFE electrodes.

## Experimental Section

### Electrode Preparation and Characterization

Porous Ti electrodes were prepared using a dry-wet spinning technique following a previously reported method.^32^ In brief, a homogeneous suspension of Ti powder with an average particle size of 6 μm (TLS Technik GmbH & Co., ASTM, grade 2), poly(ether sulfone) (PES, BASF, Ultrason E 6020P) and *N*-methylpyrrolidone (NMP, Sigma-Aldrich, ≥ 99%) is pressed through a spinneret with water as bore liquid. As-prepared fibers were thermally treated in Ar for 8 h at 800 °C to remove the polymer.^32^ Hollow fibers were connected to a Swagelok stainless steel tube using Ag epoxy glue (Chemtronics, CW2400) to allow for an electrical connection. To prevent contact of the Ag glue or the stainless steel tube with the electrolyte, an adhesive glue (Weicon, 10550024) was used to cover the contacts. Finally, the open end of the fiber was also covered with adhesive glue to enable efficient gas flow through the porous tube walls.

Ti/Ag electrodes were obtained by Ag electrodeposition.^31^ Prior to Ag electrodeposition, the samples were cleaned by cycling 10 times in the potential window 0 to −1.74 V vs RHE at a scan rate of 50 mV/s to enhance the conductivity of the surface. Ag electrodeposition was performed by potential cycling in the potential window of 0.8 to −0.5 V vs Ag/AgCl at 50 mV/s for 10 times, using an electrolyte consisting of 1 mM AgNO_3_ (Sigma-Aldrich, ReagentPlus, ≥ 99.0%) and 0.1 M KNO_3_ (Sigma-Aldrich, ≥ 99.0%). Subsequently, the potential was cycled between the nucleation (0.35 V vs Ag/AgCl) and growth potential (0.20 V vs Ag/AgCl) of Ag particles, at which the system was rested for 90 s. The Ag loading was varied by adjusting the number of cycles between the nucleation and growth potential. For a low Ag loading, deposition was performed with only one cycle (referred to as Ti/Ag-PD(1)) and higher Ag loadings were achieved using five consecutive cycles (i.e., Ti/Ag-PD(5)). Prior to any electrochemical measurement possible nitrate residues were removed by potential cycling in the potential window 0 to −1.74 V vs RHE at a scan rate of 50 mV/s. The electrodes were characterized using scanning electron microscopy and energy-dispersive X-ray spectroscopy (SEM, EDX, and ZEISS Merlin system).

### Electrochemical Measurements

**Scheme 1:**
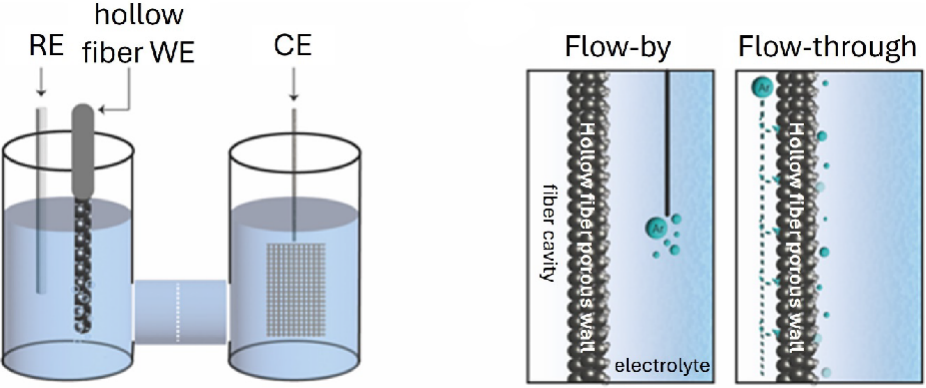
Schematic Representation of the Electrochemical H-Cell and the Two Flow Modes Used in This Study The electrodes used were Ti or Ti/Ag hollow fiber electrodes as the WE, and platinized Ti mesh and Ag/AgCl electrodes as CE and RE, respectively. The compartment volumes were 30 mL, and a Nafion 212 membrane was used to separate the two compartments.

Electrochemical experiments were performed in a H-cell using a Nafion 212 membrane to separate the compartments. The electrode properties were evaluated using 0.1 M HClO_4_ (Sigma-Aldrich, 70%) or 0.1 M HClO_4_ containing 50 mM KNO_3_ as electrolyte. A platinized Ti mesh and an Ag/AgCl (3 M NaCl, BASi, = +0.209 V vs NHE) electrode were used as counter and reference electrodes, respectively. To remove oxygen from the electrolyte, the cell was purged with Ar for at least 30 min before the experiments. The measurements were performed using continuous Ar purging utilizing the flow-through (purging through the porous walls of the hollow fiber electrode) or flow-by (conventional purging using a sparger positioned in close proximity to the electrode surface) configuration as schematically shown in Scheme 1. Cyclic voltammetry measurements were conducted at a scan rate of 50 mV/s. All experiments were performed at room temperature using a Biologic VSP potentiostat. Measured currents were converted to current densities by using the geometrical surface area of the electrodes to account for minor differences in their length. Measured potentials () were converted to the RHE scale using
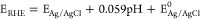
1

Additionally, the potentials reported in this work were corrected for the solution resistance (80% *iR* drop). The Faradaic efficiency toward ammonium (eq 2) and hydroxylamine (eq 3) was determined after chronopotentiometric measurements performed for 30 min by liquid phase analysis. Thus both products are denoted as liquid products.
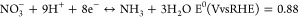
2
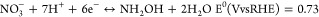
3

Note that the Faradaic efficiency to ammonium is denoted as FE_NH3_. The liquid products (ammonium and NH_2_OH) and nitrate were quantified as described in the (Figures S1–S3). The conversion of nitrate (X) and the selectivity (S) toward the liquid products were calculated using eqs 4 and 5, respectively.
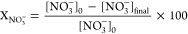
4
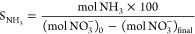

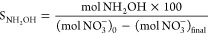
5

The sum of the selectivity toward ammonium () and hydroxylamine () is referred to as the N-selectivity_liquids_. Importantly, NO_2_^–^ was not detected as it is known to decompose rapidly in acidic media.^4,24^ During chronopotentiometric measurements, the temporal evolution of H_2_, NO, and N_2_O was revealed by mass spectrometry (see Figure S4). Note that nitrogen was not detected. Measurement errors were obtained as the standard deviation of individual measurements.

## Results and Discussion

### Characterization and Cyclic Voltammetry

**Figure 1:**
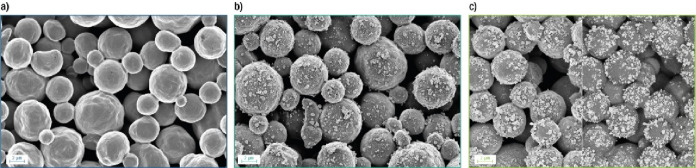
Scanning electron microscopy images of (a) Ti, (b) Ti/Ag-PD(1) and (c) Ti/Ag-PD(5) hollow fiber electrodes. As indicated by the scale bars (2 μm), all pictures have been obtained at a similar resolution.

Ag decorated Ti hollow fibers (Ti/Ag) were prepared by using the described pulsed deposition approach. The successful deposition of Ag particles at the outer surface of the Ti hollow fiber electrodes is confirmed by SEM (Figure 1) and EDX (Figure S5). Independent of the Ag loading, Ag is predominantly deposited as agglomerated particles, and for depositions using consecutive cycles, as suggested by SEM (Figure 1), a higher Ag loading is obtained. The Ag particle size distribution is wide (from ∼0.08 to ∼0.8 μm), and some branched structures are observed for Ti/Ag-PD(5) electrodes (see also Figure S5).

**Figure 2:**
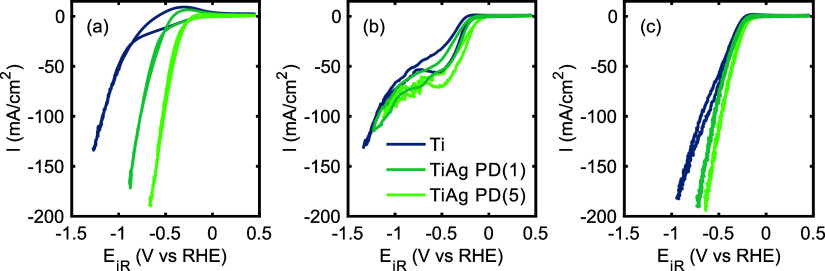
Cyclic Voltammetry measurements with Ti, Ti/Ag PD(1) and Ti/Ag PD(5) hollow fiber electrodes using (a) 0.1 M HClO_4_, and (b, c) 0.1 M HClO_4_ and 50 mM KNO_3_ as electrolyte. In (c), flow-through conditions were used at an Ar flow rate of 20 mL/min.

Cyclic voltammetry of the three electrodes performed in 0.1 M HClO_4_ (Figure 2a) or in 0.1 M HClO_4_ and 50 mM NO_3_^–^ (Figure 2b) using flow-by conditions—hence in mass transfer limited conditions—is shown in Figure 2. The onset potential for HER in the absence of nitrate of ∼ −0.80 V vs RHE for Ti electrodes (Figures 2a and S6–S8) is in good agreement with earlier reports,^23,24^ and a shift to more positive potentials is observed for both Ti/Ag electrodes. With higher Ag loading, a less negative onset potential for HER (−0.50 and −0.25 V vs RHE, respectively) is observed likely as a result of the abundance of Ag particles (see Figure 1). The presence of nitrate results in a shift of the current onset potential to more positive values. The lowest onset potential is observed for Ti/Ag-PD(5), with values of −0.20 V for Ti, −0.15 V for Ti/Ag-PD(1), and −0.05 V for Ti/Ag-PD(5), respectively. In comparison to the measurements conducted in the absence of nitrate, a mass-transport limited regime was observed in the potential region from approximately −0.45 V to approximately −0.75 V vs RHE for all electrodes tested. Indeed, a mass-transfer limited regime in electrochemical nitrate reduction has been previously reported for a range of transition metal electrodes.^17^ At potentials more negative than −0.75 V vs RHE, all three electrodes exhibit a similar current–potential behavior with an exponential increase in current. This behavior appears to be primarily determined by the characteristics of the Ti electrode rather than the Ag particles. This is evidenced by the significantly lower currents achieved for the Ag-containing HFEs in the presence of nitrate in solution suggesting that H_2_ evolution is suppressed on Ag particles but proceeds at the Ti surface. Further discussion of the suppression of hydrogen evolution in the presence of nitrate will be presented in the subsequent paragraph, which will focus on the formation of liquid products, namely, ammonia (NH_3_) and hydroxylamine (NH_2_OH).

In order to induce mixing near the electrode surface (Figure 2c), thereby increasing the mass transport of reactants (nitrate) and products (gas and liquid) at the solid-electrolyte interface, an inert gas (Ar) was flowed through the porous walls of the electrodes (flow-through conditions). The current onset potential, defined as the potential at which a significant reductive current is observed, is consistently lower in the presence of nitrate in the electrolyte, irrespective of the Ar flow rate, which serves as a measure of the degree of induced mixing. This finding is supported by the comparative presentation of the data shown in the Supporting Information (Figures S6–S9). Moreover as a consequence of the enhanced mixing at the interface, the mass transport-limited regime between approximately −0.45 V and approximately −0.75 V vs RHE associated with nitrate reduction is absent, and the exponential increase of the current as a function of increasingly negative potential extends below −0.5 V vs RHE, particularly for both Ti/Ag electrodes. It is noteworthy that the Ti electrodes display a slight divergence from the overall trend, necessitating a higher overpotential to achieve comparable current densities as observed with the Ti/Ag electrodes.

A more comprehensive examination of the influence of the gas flow rate on the I–V curve of the three electrodes in nitrate reduction is presented in Figure S6a–c. For Ti electrodes with an increasing Ar flow rate, a gradual increase in current density is observed, which suggests that transport limitations are being alleviated. In order to establish a continuous exponential increase in current with an increasingly negative potential, a flow rate of at least 20 mL/min is required for Ti electrodes. For Ti/Ag-PD(1), and even more so for Ti/Ag-PD(5), mass transfer limitations are alleviated at flow rates of 5–10 mL/min (flow-through conditions). As illustrated in Figure S7, the gas flow rate in flow-through conditions in the absence of nitrate has a negligible impact on the I–V characteristics of Ti. In contrast, for Ti/Ag-PD(1), a gradual shift toward less negative potentials of the I–V curve is observed, which differs from the behavior observed in nitrate-containing electrolyte. Based on these observations, it is reasonable to conclude that the evolution of hydrogen at both Ti/Ag hollow fiber electrodes is dependent on mass transport conditions. This is particularly evident in the case of low Ar gas flow velocities and in the absence of nitrate, where the removal of hydrogen from the surface is limiting. While the differences in mass transport alleviation are being investigated further, the flow-rate-dependent changes may be explained by variations in product distribution and transport requirements, such as the probability of bubble release and bubble size, which are dependent on the electrode material. Additionally, the impact of Ag-particle deposition on the gas-flow-dependent hydrodynamic properties is currently being examined in more detail.

In summary, cyclic voltammetry studies revealed that for Ti electrodes, the H_2_ evolution reaction characteristics are hardly affected by gas flow rate, while significant improvement in the nitrate reduction reaction is observed, confirming that H_2_ evolution is kinetically limited for Ti electrodes, and nitrate reduction suffers from low nitrate surface concentrations at the Ti electrode surface and is thus considered mass transfer limited (Figure S8a–c). For Ti/Ag-PD(1) (Figure S9a–c), H_2_ evolution and nitrate reduction are both strongly affected by gas flow rate as suggested by the I–V curves, confirming H_2_ evolution and nitrate (at none or low gas velocity) are mass transfer limited. An important observation to be made is that the mass transfer induced plateau in the nitrate reduction reaction in the absence of flow implies that in the presence of nitrate, or reduction intermediates thereof, the hydrogen evolution reaction is inhibited and particularly on Ag-containing HFEs larger overpotentials are required to stimulate H_2_ formation (see Figure S10).

### Formation of Liquid Products—–NH_3_ and NH_2_OH

**Figure 3:**
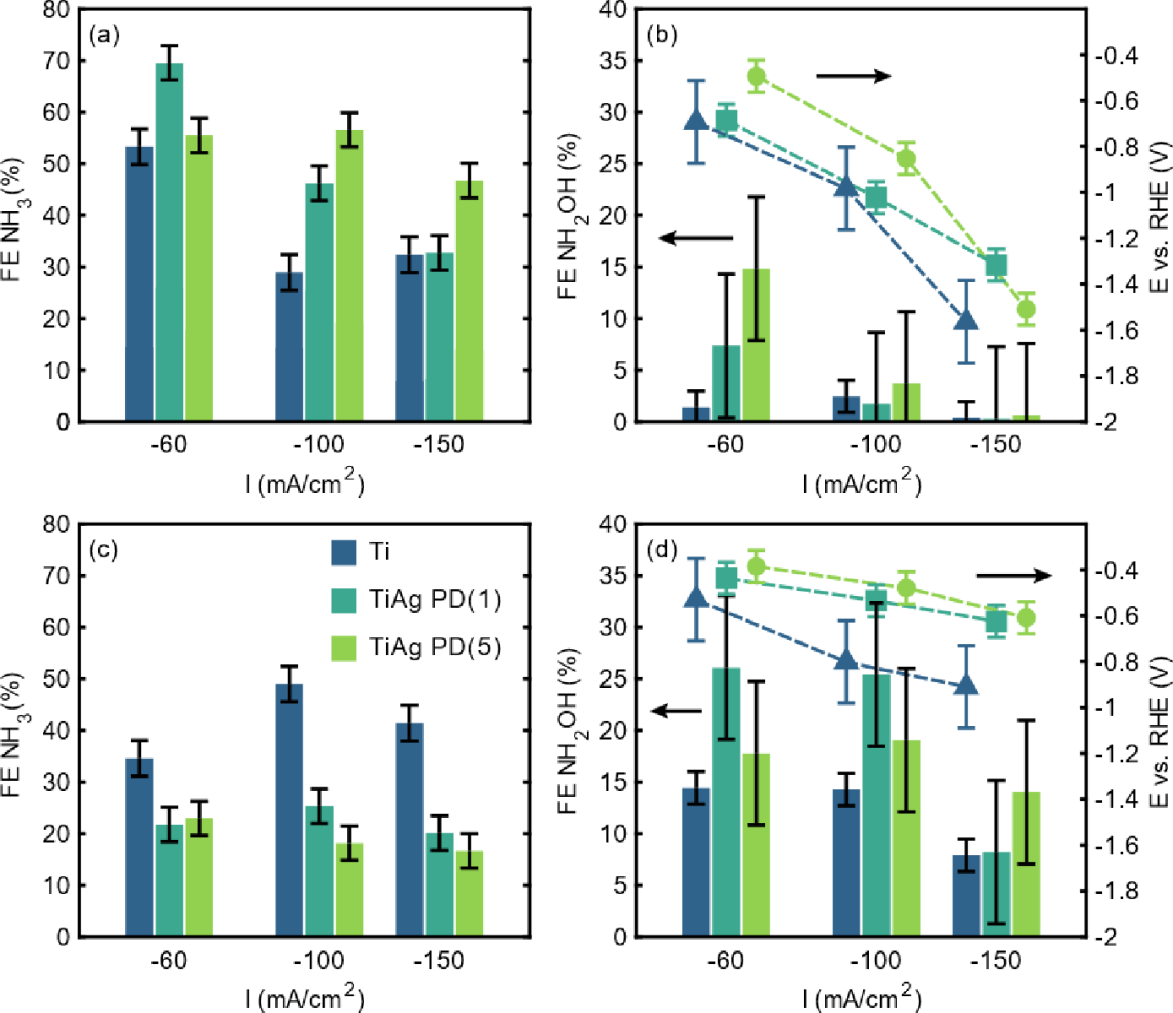
Faradaic efficiencies of ammonium and NH_2_OH determined by NMR and GC (see Supporting Information for details) after chronopotentiometry at three current densities (−60, −100, and −150 mA/cm^2^) in 0.1 M HClO_4_ with 50 mM KNO_3_ for flow-by (a, b) and flow-through (c, d) conditions using an Ar flow rate of 20 mL/min. The required applied half-cell potential to maintain galvanostatic operation at the different current densities is additionally provided (b, d).

In the following we will elaborate on the effect of flow configuration on the Faradaic efficiency toward liquid products (ammonium and hydroxylamine) using chronopotentiometry performed at current densities up to −150 mA/cm^2^ as summarized in Figure 3a,b–in the absence of a gas flow (flow-by), and in Figure 3c,d for flow-through conditions using an Ar gas flow of 20 mL/min. The corresponding production rates of ammonium and NH_2_OH are shown in Figure S10.

Although generally applicable, a more negative potential is required to operate at higher current densities (trend indicated by dashed line), being less negative for both Ag/Ti electrodes in agreement with the cyclic voltammetry measurements and also confirmed by the potential-time profiles summarized in Figure S12. Importantly, the FE_NH3_ is generally larger in flow-by conditions than in flow-through conditions (compare Figure 3a,c). Moreover, under mass transfer limited conditions in flow-by mode (Figure 3b), the FE toward NH_2_OH is low for Ti electrodes and decreasing as a function of increasing current density for both Ti/Ag electrodes with Ti/Ag-PD(5) showing the highest Faradaic efficiency to NH_2_OH of 15% at −60 mA/cm^2^.

Interestingly, the bare Ti hollow fiber electrodes, when operated in flow-by conditions (Figure 3a), exhibited a markedly lower Faradaic efficiency for ammonium compared to Ti/Ag hollow fiber electrodes when operated at −100 mA/cm^2^ (and −150 mA/cm^2^). The observed decrease in Faradaic efficiency for Ti electrodes at high current densities (at more negative potential) may be attributed to the formation of titanium hydride (TiH_*x*_) at similar potentials (−1.6 V),^33,34^ as previously indicated by cyclic voltammetry (CV) (see Figure 2a). The observed increase in FE_NH3_ at the highest current density of −150 mA/cm^2^ is within the margin of error but may also be attributed to a hydrogen-induced catalytic nitrate reduction. Nevertheless, the determined Faradaic efficiencies (FEs) are in accordance with those reported previously,^23,24^ thereby confirming the reproducibility of the preparation and utilization of Ti hollow fiber electrodes.

To explain the higher FE_NH3_ observed for both Ti/Ag hollow fiber electrodes of up to 70%, again the CVs reported in Figures S8 and S9 are illustrative, demonstrating that the presence of nitrate (and likely reduction intermediates) suppresses the H_2_ evolution reaction (see Figure S9a). Overall, limited mass transfer to the electrode interface thus appears favorable for the performance of Ag-based electrodes in facilitating the multielectron ammonium formation reaction. Still, the Faradaic efficiencies obtained with Ti/Ag electrodes are generally lower compared to previous reports using pure Ag electrodes,^28,30^ yet it is important to point out that in other reports significantly higher nitrate concentrations have been used, as well as electrolytes of higher pH.

It is to be noted that at higher current densities a higher Ag-loading appears to be beneficial in maintaining a high FE_NH3_. This phenomenon is likely to be explained by the potential applied during chronopotentiometry. For Ti/Ag-PD(5), lower potential are applied to maintain the current density, which is generally conducive to the formation of hydroxylamine. This is consistent with the elevated FE_NH2OH_ observed for Ti/Ag-PD(5) during operation at −60 mA/cm^2^. With increasing current density and higher working electrode potential, the production of hydrogen likely becomes more significant for both Ti/Ag electrodes. Yet, Ti/Ag-PD(5) remains active for nitrate reduction, as illustrated by the overall high FE_NH3_.

Contrasting flow-by conditions, in flow-through conditions at a flow rate of 20 mL/min (Figure 3c,d) a distinctively different behavior of Ti and both Ti/Ag hollow fiber electrodes in the formation of liquid nitrate reduction products is observed, similar to the strong dependence revealed in the I–V measurements. Undoubtedly using flow-through conditions has a strong effect on the applied potential required to maintain current densities that are more negative than −60 mA/cm^2^. For example, at a current density of −150 mA/cm^2^, for Ti electrodes a potential of only −0.9 V vs RHE is required (Figure 3d), corresponding to a decrease of 700 mV in comparison to “flow-by” conditions. More importantly, the FE is changing significantly when compared to flow-by conditions. At −60 mA/cm^2^, a lower FE_NH3_ is obtained, while at −100 mA/cm^2^ (and at higher overpotential), a Faradaic efficiency of approximately 50% is revealed with Ti electrodes, corresponding to a gain of approximately 20% in FE_NH3_ compared to galvanostatic flow-by experiments performed at the same current density. Furthermore, this is accompanied by an increase in FE_NH2OH_ to approximately 15%. It can be argued that, in flow-through operation, hydrogen evolution on Ti electrodes does not occur as a consequence of the lower working electrode potential, thus allowing for significant nitrate conversion and liquid product formation.

In accordance with the CV displayed in Figure 2 for both Ti/Ag hollow fiber electrodes, the observed reduction in applied potential is more pronounced than that observed for Ti electrodes, enabling operation at current densities of −150 mA/cm^2^ at −0.6 V vs RHE (Figure 3d), rather than −1.4 V vs RHE, which is the required value in flow-by conditions. However, the FE_NH3_ is markedly lower and appears to be constrained to approximately 20%, irrespective of the current density employed. To illustrate, at a current density of −150 mA/cm^2^, the FE_NH3_ decreased by 50% in comparison to the flow-by conditions. Nevertheless, FE_NH2OH_ values of up to 25% are obtained with Ti/Ag–PD (1) (Figure 3d), indicating that nitrate reduction is maintained in a flow-through operation. The observed trends clearly indicate that, upon the addition of Ag, a meticulous regulation of the induced transport is essential to facilitate the formation of ammonium which requires operation above a certain potential.^28^ At too low potentials, hydroxylamine is the favored liquid product. Moreover, the production of hydrogen or other gaseous products, such as NO, may be enhanced, potentially leading to a reduction in the formation of N-based liquid products.

**Figure 4:**
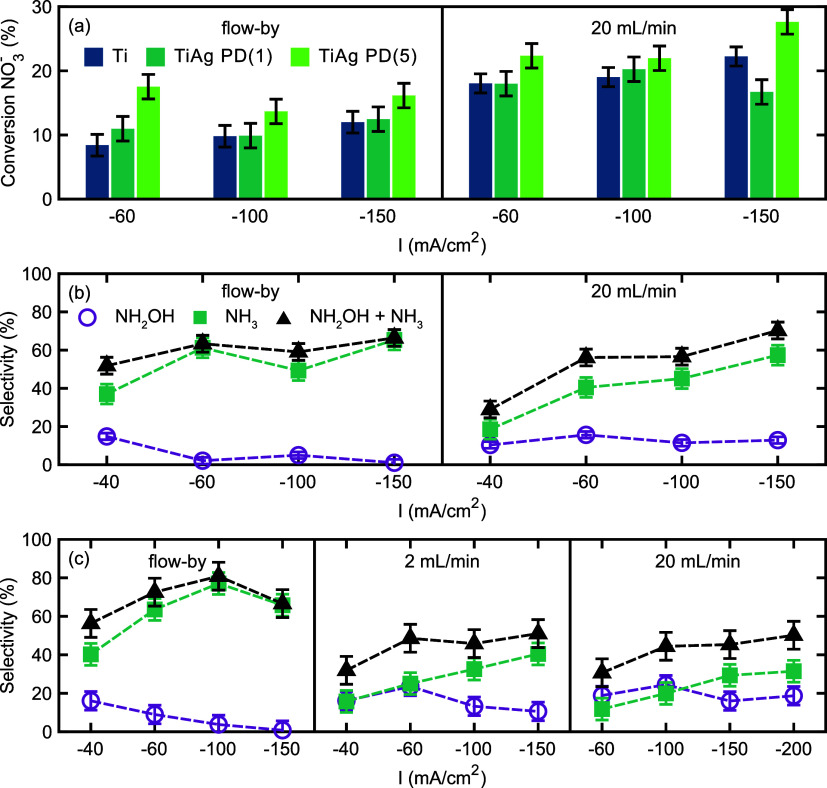
(a)Nitrate conversion after chronopotentiometry at three current densities (−60, −100, and −150 mA/cm^2^) in flow-by and (b) flow-through configuration (20 mL/min). (b) Selectivity toward liquid nitrate reduction products (NH_3_ and NH_2_OH, as well as the sum of both liquid products, i.e., the N-selectivity_liquids_) with Ti HFE and (c) Ti/Ag-PD(1) HFE in the NO_3_^–^RR calculated using eq 5 at three current densities tested in flow-by or flow-through conditions. Ar flow rates are indicated in the panels.

To gain further insight into the role of gaseous (nitrate) reduction products and to further elucidate the differences in material properties and transport phenomena, the conversion of nitrate has been determined. Figure 4 summarizes the conversion of NO_3_^–^ for the different electrodes and process conditions used. In flow-by conditions X_NO3_ is hardly dependent on current density and appears to be limited to 8 to 15% with Ti/Ag-PD(5) being most efficient in nitrate conversion highlighting the more significant role of hydrogen evolution with increasing current densities. In flow-through conditions, nitrate conversion is more favorable agreeing with an improvement in transport of reactants that is required for higher nitrate conversion.^24,35−37^ Nitrate conversion for Ti/Ag electrodes is ambiguous–some improved nitrate conversion is observed at −150 mA/cm^2^ for Ti/Ag-PD(5) in comparison to the Ti electrodes, while for Ti/Ag-PD(1) and at −150 mA/cm^2^ the conversion is lower. Overall, the nitrate conversion remains at approximately 20%, again with Ti/Ag-PD(5) being most efficient reaching X_NO3_ of up to 25%. This generally agrees with earlier reports showing that Ag enables efficient conversion of nitrate.^17^ It is assumed that the difference in morphology and silver loading between the two Ti/Ag hollow fiber electrodes, and associated hydrodynamic behavior, causes the small differences in nitrate conversion.

Considering the changes in FE toward liquid products and the changes in X_NO3_, we suggest that in flow-through conditions at lower potentials conversion of NO_3_^–^ to NO_2_^–^ occurs, in agreement with earlier reports,^28^ followed by partial consecutive reduction to NH_2_OH at Ti/Ag electrodes. Local mixing at the Ti/Ag interface likely provokes effective removal of NO_2_^–^ (and/or its decomposition product NO), NH_2_OH and other NO_*x*_ products, preventing consecutive hydrogenation to ammonium. Efficient removal of intermediates and products likely also reduces surface poisoning and enables H_2_ formation (see Figure S9)—both explaining the low FE toward ammonium determined for Ti/Ag electrodes (see Figure 3). Nevertheless, it has to be noted that changes in nitrate conversion and product distribution also have a direct impact on the local pH at the surface of the electrode, which is thus important to be considered in this context. The increase in nitrate conversion additionally suggests that for Ti electrodes operated in flow-through conditions at high current densities (more negative than 60 mA/cm^2^) hydrogen evolution is sufficiently suppressed in agreement with the data presented in Figure S8.

Finally, the selectivity toward liquid nitrate reduction products was calculated according to eqs 4 and 5. For Ti electrodes (Figure 4c), in the absence of flow (left), the NH_2_OH selectivity (S_NH2OH_) is low and even decreases when higher current densities are applied. Simultaneously, an overall increase in S_NH3_ to >60% is observed. In flow-through conditions, the S_NH2OH_ remains constant for all current densities tested, but a clear rise in the ammonium selectivity to values only slightly lower than in the absence of flow is obtained. Considering that the X_NO3_ remains constant, the increase in ammonium selectivity is directly related to an efficient conversion of intermediates, i.e., the two-electron reduction product nitrite. Overall, for Ti electrodes operated in flow-through and flow-by conditions, S_NH3_ and S_NH2OH_ account for an N-selectivity_liquids_ (see gray dashed SUM traces in Figure 4c) of >65% at the highest current densities used in this study.

In flow-by conditions, the NH_2_OH selectivity for Ti/Ag-PD (1) (Figure 4d) continuously decreases as a function of increasing current density. Conversely, the ammonia selectivity exhibits an increase in selectivity with current density up to a current density of −100 mA/cm^2^. The N-selectivity liquids (Figure 4d) in flow-by mode maintain a N-selectivity of above 70% at current densities of −60 and −100 mA/cm^2^. As previously discussed, the reduction of NH_2_OH to NH_3_ is more selective with increasing overpotentials. This is probably induced by a growing diffusion layer, which reduces the surface concentration of nitrate, as previously explained.^28^ In flow-by mode at current densities more negative than 100 mA/cm^2^, however, the total selectivity to liquid products decreases to approximately 60% for Ti/Ag-PD(1), thus being similar to the N-selectivity_liquids_ obtained with Ti electrodes used in flow-through conditions. This may be indicative of significant limitations in reactant diffusion with an increasing contribution from the hydrogen evolution reaction in the case of Ti/Ag-PD(1). In accordance with the aforementioned discussion, utilizing Ti/Ag-PD(1) under flow-through conditions is evidently detrimental to the formation of liquid nitrate reduction products. This is a consequence of the operation at a low potential, which facilitates the formation of nitrite or gaseous nitrate reduction products, rather than the formation of products that require multiple subsequent electron transfer steps. This is again in accordance with the selectivity trend observed, which reveals that the sum of NH_2_OH to NH_3_ is continuously increasing with applied current density, while nitrate conversion remains unaffected.

Contrary, flow-through conditions overall enhance the performance of Ti electrodes particularly at higher current densities. Ti electrodes are generally considered to be selective for ammonia formation over other nitrate reduction products and have been reported to follow a NO* dissociation mechanism thereby favoring ammonia over hydroxylamine formation.^17^ Thus, for Ti electrodes operated at high current densities and consequently at sufficiently large overpotentials (<−0.8 V vs RHE), the reaction mechanism, following the dissociation of NO* into N* and O* and subsequent hydrogenation of adsorbed N* by H* in a Langmuir–Hinshelwood mechanism to ammonium, ensures selectivity toward ammonium even in flow-through conditions that closely resemble the ammonium selectivity observed in flow-by conditions for Ti and Ti/Ag-PD (1). As shown in Figure 3, in flow-through conditions at high current density, it is even feasible to obtain a higher Faradaic efficiency to ammonium.^24,28,37^

Considering the detrimental influence of enhanced local mixing observed under flow-through conditions for Ti/Ag-PD (1), it appears important to understand the impact of Ar flow and induced local mixing in more detail. To tailor the extend of local mixing, flow-through experiments using an Ar flow rate of only 2 mL/min (being the smallest addressable flow rate) were performed (see Figure 4d and also Figure S9). Indeed a higher FE_NH3_ of approximately 40% was obtained at lower overpotentials being considerably larger than the FE_NH3_ obtained at high flow rates (25.3%). Moreover, the Faradaic efficiency to hydroxylamine in flow-through conditions (2 mL/min) exceeds the Faradaic efficiency obtained in flow-by mode operation, highlighting the complex interplay between mass transport and product selectivity, and suggests that fine-tuning of porosity of the Ti hollow fiber electrode and precise control of Ar flow rates will enable ammonium formation with high Faradaic efficiency and at low overpotentials.

To substantiate the discussion above and in order to close the overall nitrogen balance (being lower than 100% when considering only liquid products), gaseous products formed during the NO_3_^–^ RR when using Ti and Ti/Ag electrodes were analyzed by mass spectrometry. Particularly, H_2_ (*m*/*z* = 2), NO (*m*/*z* = 30) and N_2_O (*m*/*z* = 44) were observed using flow-by conditions (see Figure S4 for a schematic of the cell operation in flow-by mode). The gas analysis (Figure S12) shows similar time dependent trends in the intensity of the recorded mass over charge signals irrespective of the electrode. At a low current density, the main gaseous products are NO and N_2_O, while at higher current density primarily H_2_ is produced, to a much greater extent for Ti than for the Ti/Ag-PD (1). This is in agreement with the observed inhibition of the HER in the presence of nitrate, the higher Faradaic efficiency for ammonium (Figure 3a) and the high N-selectivity_liquids_ (Figure 4d) for Ti/Ag-PD(1) when operated in flow-by mode. Most importantly, qualitatively, the gas analysis confirms the formation of NO and N_2_O, which thus likely accounts for closure of the overall nitrogen balance.

In order to provide a comprehensive overview of the experimental observations and hypotheses, it is beneficial to summarize them in a structured manner. The data indicate that in the flow-by conditions, a relatively high concentration of reduction intermediates, particularly, prevents extensive formation of H_2_ even at strongly negative polarization and instead facilitates the formation of ammonium when Ag is deposited on the Ti hollow fiber electrodes. An Ar-flow through the porous walls of the Ti electrode of Ti/Ag HFE generally stimulates the transport of intermediates away from the electrode surface, leading to higher concentrations (and FE) of NH_2_OH and NO (either formed via spontaneous decomposition of NO^2–^^24^ or as a general reduction intermediate). This diminishes the FE_NH3_. Notwithstanding the aforementioned implications regarding ammonium formation on Ti/Ag HFE, this work additionally reveals that ensuring the efficient transport of reactants to the Ti surface in Ti HFEs is crucial for suppressing parasitic H_2_ formation and facilitating nitrate conversion. In conclusion, the implementation of an efficient transport mechanism, as observed in flow-through mode, facilitates ammonium formation at Ti HFEs at rates comparable to those observed in Ti/Ag HFEs in flow-by mode. However, this is achieved at considerably lower overpotentials and with an overall higher nitrate conversion. Therefore, optimizing the hydrodynamic properties represents an effective approach to induce selective conversion of nitrate at Ti hollow fiber electrodes, particularly and at Ti electrodes in general, without the necessity for the addition of any costly materials.

## Conclusions

In this study, we stress the influence of reactant and product transport in electrochemical nitrate reduction using Ti and Ti/Ag hollow fiber electrodes using galvanostatic process control. Local mixing of the electrolyte near the solid–electrolyte interface is shown to have a strong electrode material dependent influence on the activity and selectivity in the NO_3_ RR. The modification of Ti hollow fiber electrodes with Ag particles was shown to improve the Faradaic efficiency and selectivity to ammonium in diffusion limited conditions. Nitrate and reduction intermediates thereof suppress the evolution of H_2_ under these conditions. However, when introducing efficient electrolyte-mixing near the Ag electrode surface the ammonium selectivity decreases, though the overall nitrate conversion is increasing. Importantly for pure Ti electrodes, due to their inherent poor activity for the HER (confirmed by the blank experiments in HClO_4_), local mixing results in a higher Faradaic efficiency to ammonium particularly at high current densities, outperforming Ti/Ag electrodes in terms of required polarization potential (600 mV gain compared to flow-by conditions) and ammonium formation. This study thus suggest that detailed understanding of the performance of electrocatalytic surfaces in nitrate reduction should go hand in hand with understanding of mass transport related phenomena, already in early stages of materials development.

## Supporting Information Available

Experimental details including calibration data, additional experimental data including cyclic voltammetry, mass spectrometry, scanning electrochemical microscopy, and chronopotentiometry measurements (PDF)

## Abbreviations

NO_3_ RR: nitrate reduction reaction
HER: hydrogen evolution reaction
EC-MS: electrochemical–mass spectrometer
HFE: hollow fiber electrode
FE: Faradaic efficiency
