# Supplementary material for: Role of Mass Transfer Phenomena in Electrochemical Nitrate Reduction: A Case Study Using Ti and Ag-Modified Ti-Hollow Fiber Electrodes
Source: ACS Eng Au. 2024 Dec 24;5(1):27–35. doi: 10.1021/acsengineeringau.4c00035 (PMC11843601; doi:10.1021/acsengineeringau.4c00035)
Supplement: Supplementary file 1 — eg4c00035_si_001.pdf [file eg4c00035_si_001.pdf]

Supporting Information:

On the role of mass transfer phenomena in electrochemical nitrate reduction: A case study using Ti and Ag-modified Ti-hollow fiber electrodes

Ainoa Paradelo Rodríguez,[a] *Guido Mul,*[a,*] *Bastian T. Mei*[a,b,*]

[a] Photocatalytic Synthesis Group, Faculty of Science & Technology of the University of Twente, PO Box 217, Enschede, The Netherlands

[b] Technische Chemie, Ruhr-Universität Bochum, Universitätsstr. 150, 44801 Bochum, Germany

Ammonia quantification

Ammonia quantification was determined using a Bruker 400 MHz NMR spectrometer with 1000 scans by employing 1H NMR spectroscopy based on a previously reported method[1] (Figure S2a). Calibration curves were obtained using the standard solution of a known concentration of NH_4Cl. Standard solutions were prepared by mixing the respective amount of NH_4Cl with 50 μL of 0.5 M H_2SO_4 which contained 10 mM of maleic acid used as an internal reference, and 25 μL of DMSO-d6 used as solvent. A linear regression was obtained using the ratio of the peak area of NH_4^+ and maleic acid plotted against NH_3 concentration (Figure S2b).

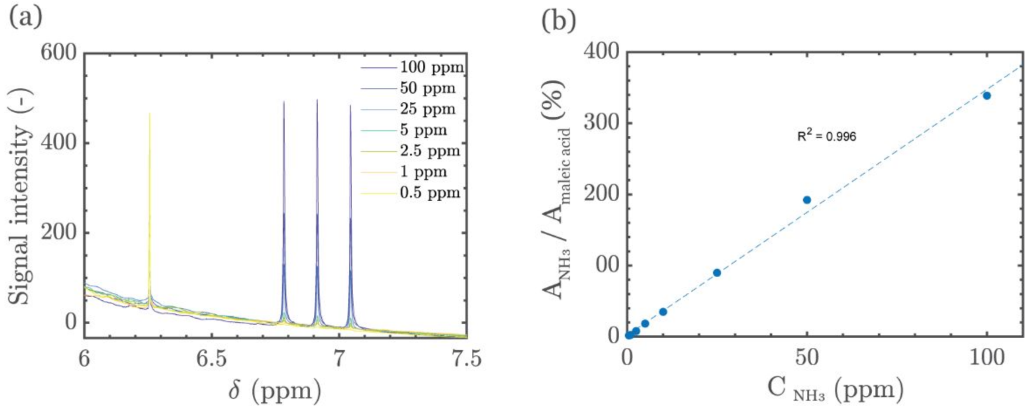

Figure S1. (a) ^{1}H NMR spectra obtained for the different concentrations of NH_4^+ as indicated in the plot and (b) linear regression used as calibration curve for quantification of NH_4^+.

Hydroxylamine quantification

Hydroxylamine was detected as acetone oxime using a flame ionization detector (GC- FID, Aligent gas chromatography) on a Zebron 7HG-G013-11 (0.25μm ID, 250 μm, 40m) column following a previously

reported method [2]. To obtain acetone oxime, 4 mL of the samples were neutralized by addition of 1 M of NaOH solution. Afterwards, 5 µL of a methanol/acetone (1:1, v/v) solution was added to form acetone oxime. Standard solutions of hydroxylamine hydrochloride were used to obtain the calibration curve. Linear regression was used to obtain the calibration curve from the integrated area of the respective GC-FID peaks versus the known concentration of hydroxylamine (Figure).

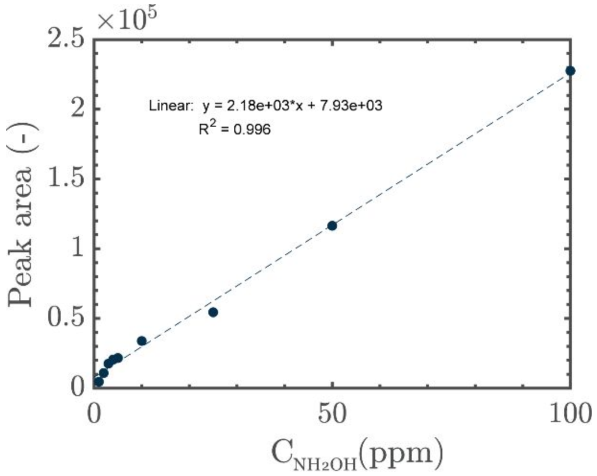

Figure S2. Integrated peak area against the known concentration of hydroxylamine. The calibration curve for NH_2OH quantification was obtained using linear regression.

Nitrate (NO_3^-) quantification

Nitrate was detected by ion chromatography (IC) using the ICS-3000 device from Thermo Fisher Scientific. The anion eluent used was 100 mM KOH and the cation eluent was 20 mM MSA.

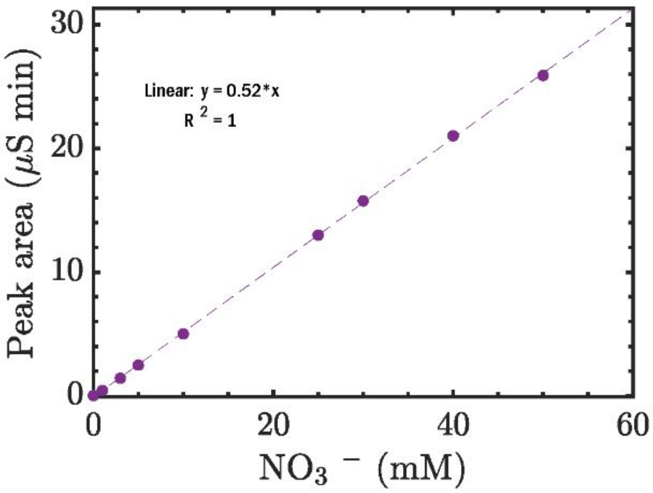

Figure S3. Integrated peak area against the known nitrate concentration and the corresponding linear regression used as calibration curve for NO_3^- quantification.

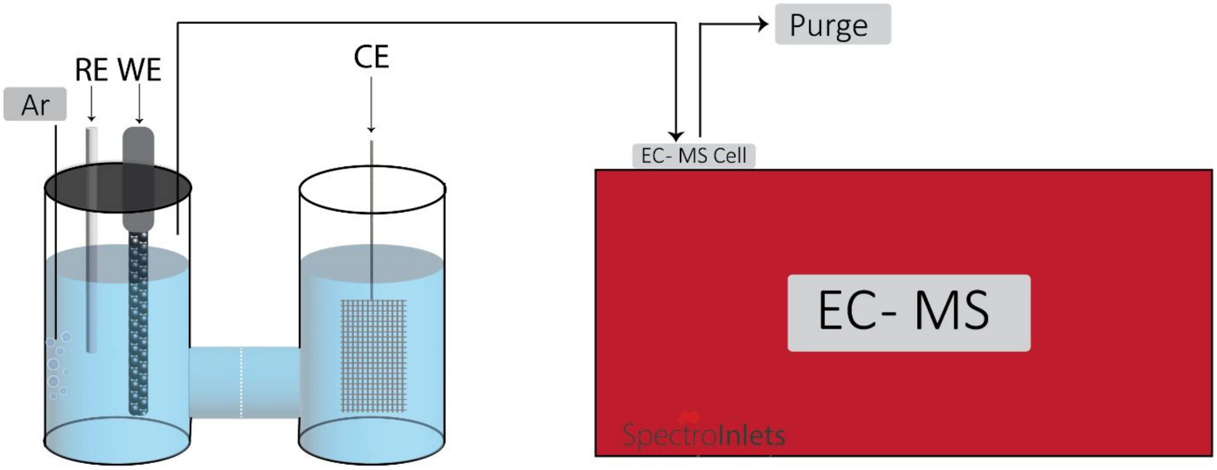

Figure S4. Schematic representation of H-Cell connected to the EC-MS[3] for gas analysis.

NO_3^- electroreduction reactions

Table S1. Standard reduction potentials of NO_3^- electroreduction reactions.

Reaction	$\boldsymbol{E^0}$ ($\boldsymbol{V\ vs\ RHE}$)
$NO_3^- + 2H^+ + e^- \leftrightarrow N0_2 + H_2O$	0.77
$NO_3^- + 2H^+ + 2e^- \leftrightarrow NO_2^- + H_2O$	0.94
$NO_3^- + 4H^+ + 3e^- \leftrightarrow NO + 2H_2O$	0.96
$2NO_3^- + 10H^+ + 8e^- \leftrightarrow N_2O + 5H_2O$	1.12
$2NO_3^- + 12H^+ + 10e^- \leftrightarrow N_2 + 6H_2O$	1.25
$NO_3^- + 7H^+ + 6e^- \leftrightarrow NH_2OH + 2H_2O$	0.73
$2NO_3^- + 16H^+ + 14e^- \leftrightarrow N_2H_4 + 6H_2O$	0.82
$NO_3^- + 9H^+ + 8e^- \leftrightarrow NH_3 + 3H_2O$	0.88

Electrode characterization

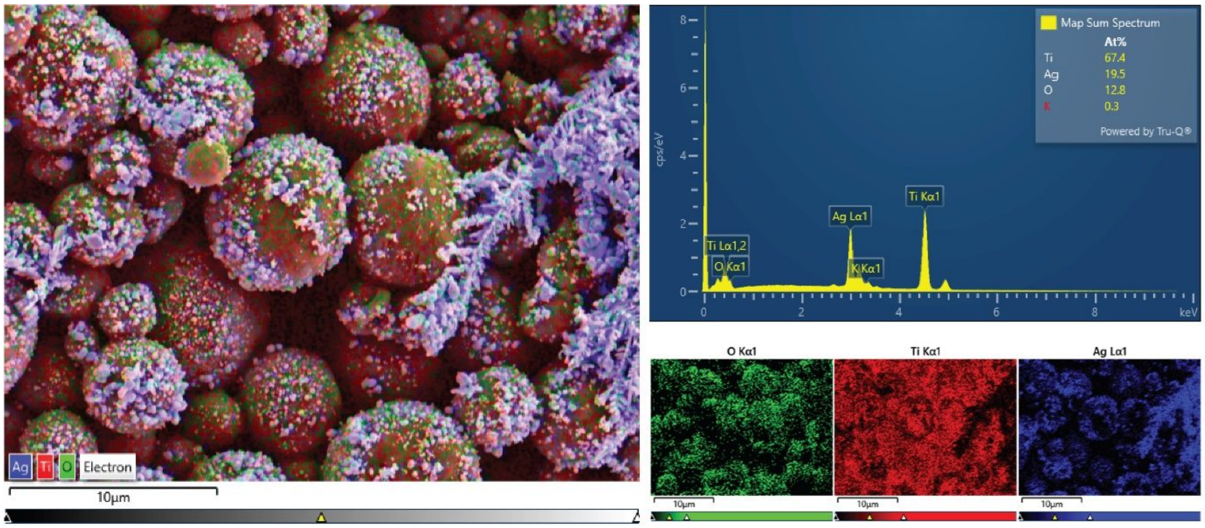

Figure S5. SEM picture and corresponding EDX data of the Ti hollow fiber surface decorated with Ag particles obtained by electrodeposition. Here the TiAg – PD(5) hollow fiber electrode is shown. Left: SEM picture and overlayed EDX data to highlight the Ag particle distribution; right EDX spectrum and color code maps of oxygen (green), titanium (red), and silver (blue).

Influence of flow rate

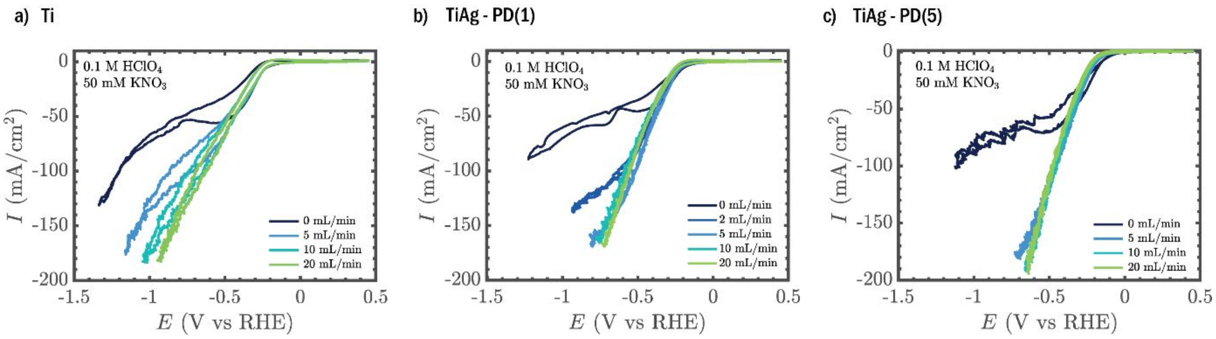

Figure S6. Cyclic Voltammetry measurements obtained with (a) Ti, (b) TiAg – PD(1) and (c) TiAg – PD(5) hollow fiber electrodes using different flow rates performed at 50 mV/s in 0.1 M $HClO_4$ and 50 mM KNO_3 solutions. The CE and RE were platinized Ti mesh and Ag/AgCl, respectively. The results highlight the dependence of the transient current-voltage behavior with the Ar flow rate. For samples containing Ag a large influence is noticeable at low flow rates with little changes at higher flow rates, whereas for Ti with increasing flow rate a progressive shift to lower potentials is observed.

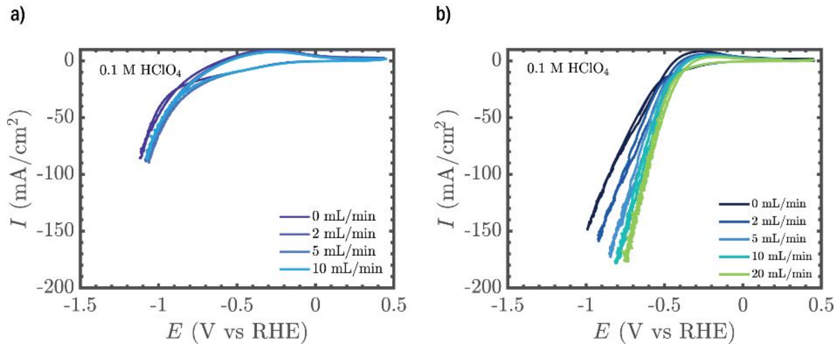

Figure S7. Cyclic Voltammetry measurements at 50 mV/s varying the flow rate on (a) Ti and (b) TiAg – PD(1) hollow fiber electrodes in 0.1 M $HClO_4$ electrolyte. The CE and RE were platinized Ti mesh and

Ag/AgCl, respectively. A small influence is observed for Ti electrodes, whereas with increasing flow rate a progress shift towards smaller potentials is obtained with TiAg electrodes.

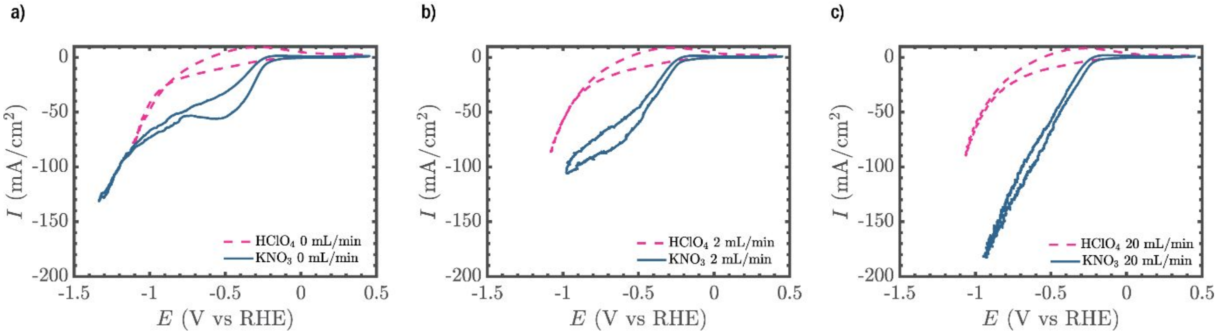

Figure S8. Cyclic Voltammetry measurements on Ti hollow fiber electrode using (a) flow-by (0 mL/min), and flow-through conditions with Ar flow rates of (b) 2 mL/min and (c) 20 mL/min using either 0.1 M $HClO_4$ (dash line) or solutions containing 0.1 M $HClO_4$ and 50 mM KNO_3 (solid line as electrolyte). All measurements are performed at a scan rate of 50 mV/s. The CE and RE were platinized Ti mesh and Ag/AgCl, respectively. Note that the HER and NO_3 RR does not overlap.

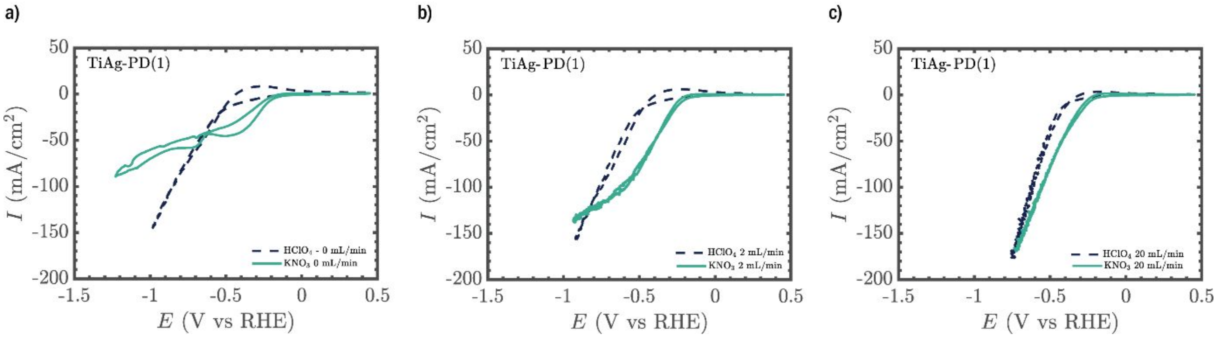

Figure S9. Cyclic Voltammetry measurements TiAg PD(1) using (a) flow-by (0 mL/min), and flow-through conditions with Ar flow rates of (b) 2 mL/min and (c) 20 mL/min. All measurements are performed at a scan rate of 50 mV/s in 0.1 M $HClO_4$ (dash line) or a solution containing 0.1 M $HClO_4$ and 50 mM KNO_3

(solid line). The CE and RE were platinized Ti mesh and Ag/AgCl, respectively. Note that there is a cross-over point of the CV in presence of nitrate with the curve for HER.

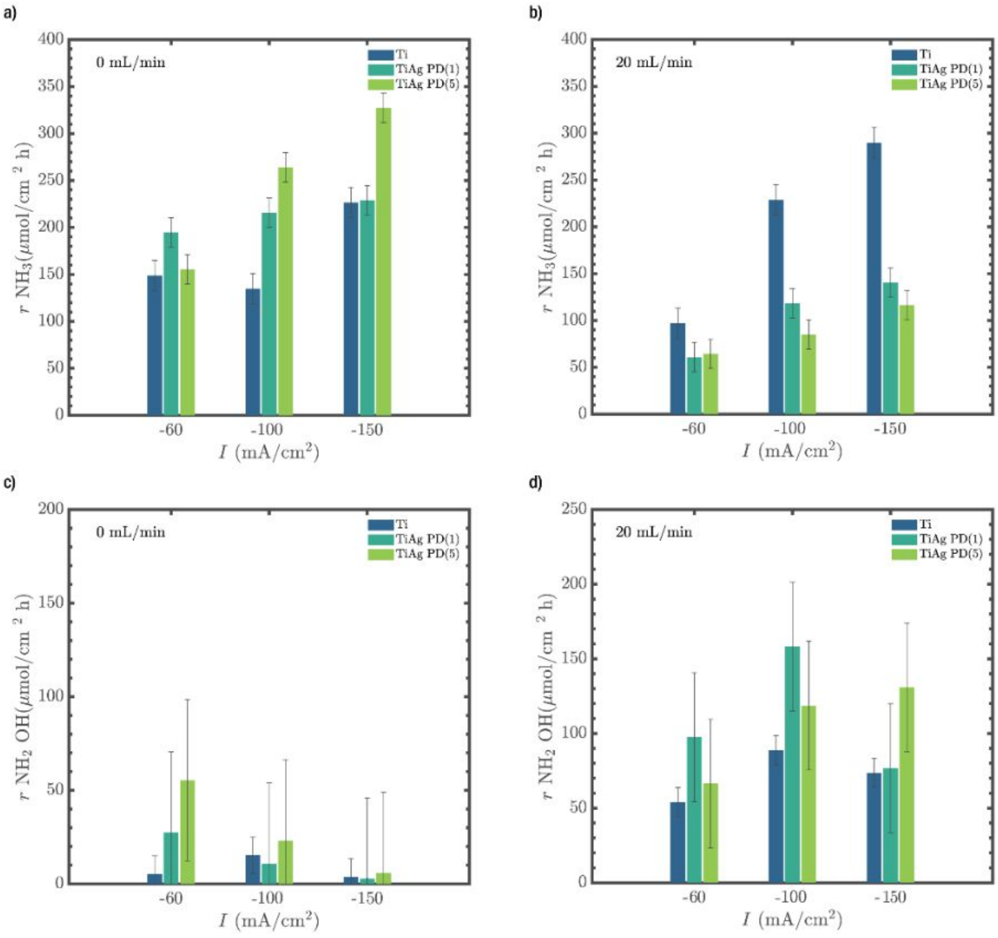

Figure S1. Production rate of NH3 and NH2OH. In flow-by (a, b) and flow-through configuration at an Ar flow rate of 20 mL/min (c, d) Rates are obtained by product quantification after chronopotentiometry at three current densities (- 60, - 100 and – 150 mA/cm^2) performed for 30 minutes in 0.1 M $HClO_4$ with 50 mM KNO_3.

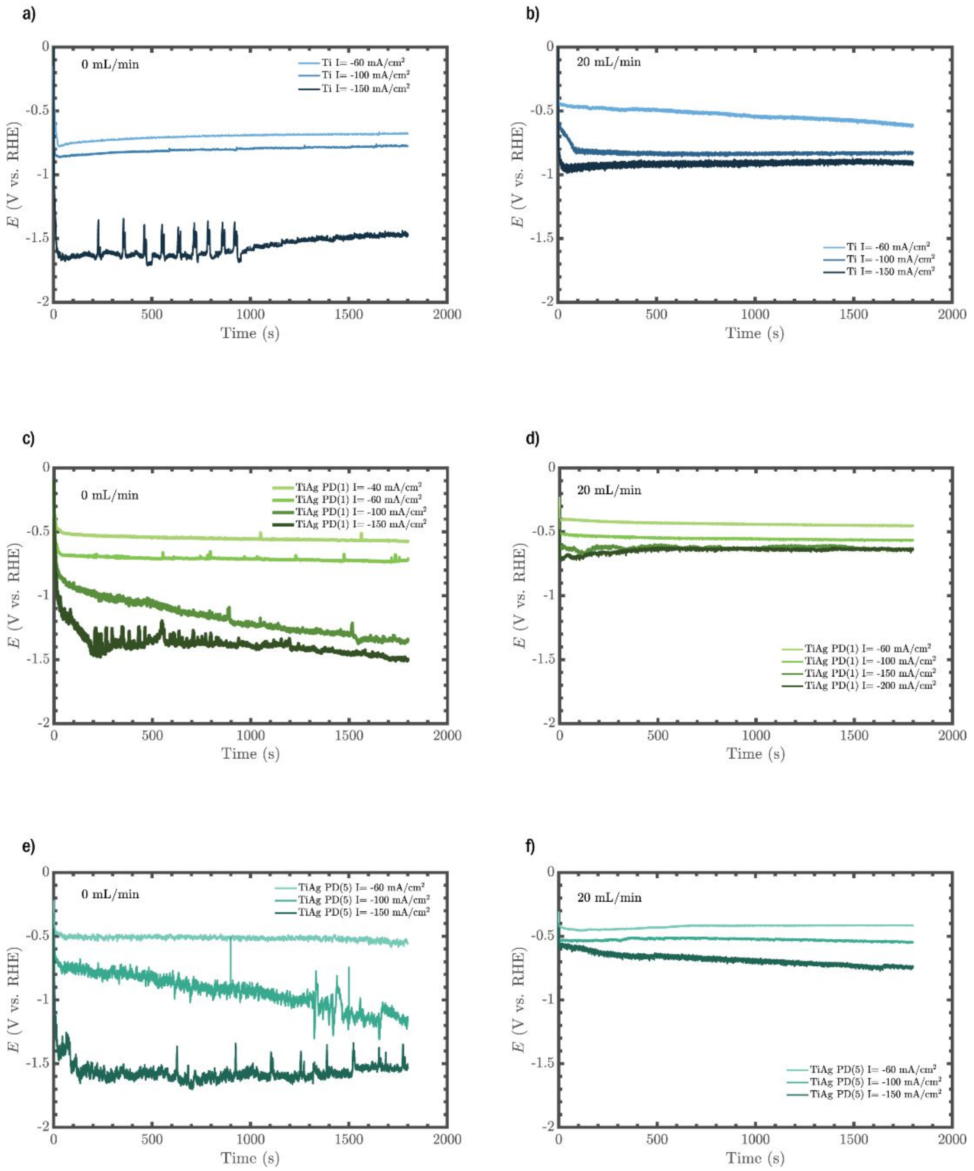

Figure S2. Potential-time profiles obtained by chronopotentiometry measurements at different current densities (as indicated in the figures) using Ti (a, b), TiAg – PD(1) (c, d) and TiAg – PD(5) (e, f) hollow fiber electrodes in flow-by (left) and flow-through configuration at a constant flow rate of 20 mL/min (right), respectively. All measurements were performed for 30 minutes using a 0.1 M $HClO_4$ with KNO_3 as electrolyte.

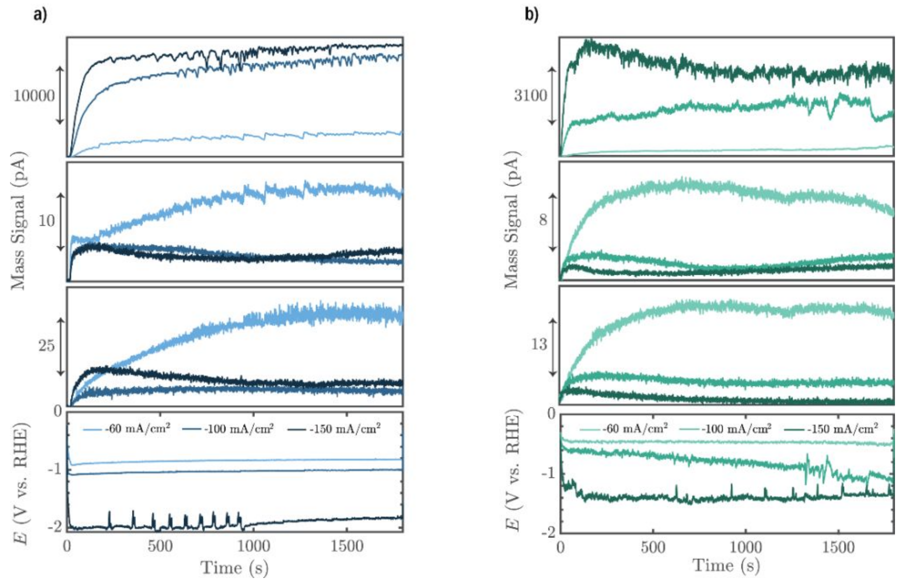

Figure S3. EC-MS measurements of (a) Ti and (b) TiAg-PD(1) electrodes using the flow-by mode configuration. In the upper panels the mass-to-charge ratio of the detected volatile products of interest (top: H_2, middle: NO, bottom: N_2O). The bottom panel shows the potential-time dependence determined by chronopotentiometry measurements performed at the current densities indicated in the plots.

References

[1] Hodgetts, R. Y. et al. Refining Universal Procedures for Ammonium Quantification via Rapid 1H NMR Analysis for Dinitrogen Reduction Studies. ACS Energy Lett 5, 736–741 (2020).

[2] Darke, D. J. Method for the measurement of hydroxylamine in colonic fluid using derivatisation and gas chromatography. J Chromatogr 181, 449–452 (1980).

[3] Trimarco, D. B. et al. Enabling real-time detection of electrochemical desorption phenomena with sub-monolayer sensitivity. Electrochim Acta 268, 520–530 (2018).
